# Distanced behind the mask: The use of non-verbal communication when counselling the elderly during the COVID-19 pandemic

**DOI:** 10.4102/hsag.v26i0.1665

**Published:** 2021-11-26

**Authors:** Mary Ann Jarvis, Lourett Smith

**Affiliations:** 1 School of Nursing and Public Health, College of Health Sciences, University of KwaZulu-Natal, Durban, South Africa; 2 School of Clinical Care Sciences and Medicinal Sciences, Faculty of Health Sciences, Nelson Mandela University, Gqeberha, South Africa

**Keywords:** elderly, counselling, COVID-19, mask, mental healthcare nurse, non-verbal communication, practice

## Abstract

The stressors caused by the coronavirus disease 2019 (COVID-19) pandemic have influenced both the physical and the mental health of the elderly, increasing their vulnerability. Counselling by the mental healthcare nurse is a critical protective factor in mitigating the mental health effects of COVID-19. However, counselling is unintentionally interrupted by the effects of the mask as a barrier to non-verbal communication. This commentary aims at conscientisation of mental healthcare nursing practice interruptions to non-verbal communication, brought upon by the pandemic. Practice recommendations focus on mask-wearing becoming less of a stressor to offset the challenges experienced by both the elderly and the mental healthcare nurses.

**Contribution:**

The authors conclude that a need exists to revisit the fundamentals of counselling, and show initiative to addressing the practice challenges created by the wearing of masks yet simultaneously contribute to #flatten_the_mental_illness_curve.

## Changes in the counselling landscape for the elderly in the realm of COVID-19

As mental health care nurses (MHCNs), guided by Zubin and Spring’s (1977) stress vulnerability model, we are acutely aware of the coronavirus disease 2019 (COVID-19) pandemic as a stressor influencing both the physical and the mental health of the elderly, increasing their vulnerability. The stress vulnerability model describes three critical factors that influence the emergence of symptomatology, namely: stressors, protective factors and vulnerability (Anderson, Ramo & Brown 2006). A year ago, in 2020, global protective initiatives to curb the spread of the virus in the elderly involved restrictions of movements (CDC 2020; WHO 2020), which appeared counter to their mental health needs. The restrictions were protective in nature, but highlighted the elderly’s psychosocial vulnerability contributing to loneliness, anxiety, depression and accelerating dementia (Carbone 2020), which created a need for counselling (Galea, Merchant & Lurie 2020). Counselling, a further protective factor, is critical in mitigating the mental health effects of COVID-19 (Schlögl & Jones 2020), and in so doing, attempts to flatten the mental-illness curve. However, we as MHCNs in the mental healthcare facilities experienced a challenge in executing our roles as counsellors to the elderly. The beneficent intent of the World Health Organization (WHO) guidelines and the South African Government COVID-19 regulations (SA Gov 2020) is to decrease the contagion of the virus through, inter-alia, the physical barrier of mandatory mask-wearing. However, an unintended negative barrier manifested in the interruption of non-verbal communication.

Mask wearing is familiar to nurses, and infection control is not a new practice (Karimi & Alavi 2015). However, the context was unfamiliar and initially novel for people not in health care. As unusual as it was for us as MHCNs to wear a mask while counselling, it was strange for the elderly. At the start of the pandemic, as MHCNs, we functioned in a trial and error, survival mode, similar to the uncertainty described by nurses in other specialities (Nelson, Hubbard Murdoch & Norman 2021). As we began counselling the elderly, our practice could be likened to unconscious incompetence as described in the stages of learning (Peel & Nolan 2015). We did not consider or reflect on the mask’s influence on psychosocial counselling and the need for practice changes. As the pandemic continued, through reflections-on-actions (Fitzgerald 1994), while we were counselling the elderly with both parties wearing masks, we became conscious of what was challenging our previously considered level of competency. In this commentary, we aim to highlight and conscientise fellow MHCNs and other mental healthcare practitioners about a practice interruption brought upon by the pandemic, hoping that it will generate further practice-related reflections.

## Challenges experienced in the interruption of non-verbal communication

Instead of being located as a protective factor, the mask’s presence shifted to a stressor creating challenges in our initial contact with the elderly. Our former welcoming smiles were hidden behind the mask. Smile intensity plays a significant role in conveying warmth, respect and competence (Padhy, Rina & Sarkar 2020), facilitating socially meaningful actions such as trust (Ames et al. 2011), a crucial component of a successful counselling session. In addition to the obscured smile, the lip movements of the MHCN are concealed, which prior to mask-wearing allowed for the elderly’s confirmation of possibly missed content because of hearing impairments. In addition, at times, we were unsure if the ensuing silence in the interaction was as result of the elderly missing what was said, and unable to rely on usual aids to interpretation, or needing time to respond. The mask, which often serves as a muffler of sound and a barrier to further lip-reading, which would typically allow for the interpretation of less audible sounds, can undermine the development of relationships (Schlögl & Jones 2020). Both the elderly and MHCNs seek to look at facial expressions to gauge the counselling session’s direction.

We found that we increased our voice volume in an attempt to be heard, which we realise posed a risk for the misimpression of inequitable power. Power discourses, synonymous with dominance and abuse, resonate with concern from studies targeting communication in the elderly (Kwame & Petrucka 2020). During the pandemic, couched in mandatory mask-wearing, power imbalances are an undesirable outcome of interactions, and we were concerned it would hinder the elderly’s personal agency of their mental health.

Apart from the more obvious thwarted counselling needs of the elderly, brought about by power and hearing impairments, we were less mindful of the mask’s negative influence on the elderly with neurocognitive decline. In the presence of neurocognitive decline, when the lower half of the MHCN’s face is concealed, the sense of disconnect and the complexity of counselling are increased, as dependence lies on additional facial cues to assist in interpreting conversations (Vaidhyanathan et al. 2020).

## Recommendations for mental health nursing practise

Our recommendations are centred on the mask becoming less of a stressor and barrier to practise delivery in that non-verbal communication becomes less strained and offers its protective value. Reflecting on our actions and examining our restricted use of non-verbal communication skills with the vulnerable elderly, we considered how to deliver empathic, relevant and responsive mental healthcare, with half of our faces concealed. The MHCN needs to become more reliant on other forms of non-verbal communication, while remaining cognisant of factors such as the pandemic’s regulations, the ageing process, and cultural sensitivity.

In response to no longer being able to use a welcoming smile to dissipate anxiety and communicate warmth, we shifted to increased use of the upper half of the face and hand movements. In considering the voice tone, pace, personal reflections, we asked: ‘Am I too loud? Am I starting to sound bossy?’ Vaidhyanathan et al. (2020:2) have provided valuable practical practise suggestions and have tabulated strategies to aid in effective communication.

The strategies suggested by Vaidhyanathan et al. (2020:2) include, inter alia, speech modifications (‘clear pronunciation, talk louder, exaggerate intonation, stress on important words’), cognitive linguistic strategies (‘active listening, summarising and chunking, checking/feedback, repetition/paraphrasing’), and environmental modifications (‘ensure speaker visibility, reduce background noise’). The necessity to reduce background noise is not a foreign concept (Vaidhyanathan et al. 2020; Xyrichis et al. 2018) and holds significance considering the context of the elderly being counselled in settings with unbaiting noise and the added restraint of the mask. In addition to speaking clearly, a need exists for the MHCN to speak slowly as diminished visibility of the non-verbal decreases the processing of the communication (Vaidhyanathan et al. 2020). Further, Mehta, Venkatasubramanian and Chandra (2020) suggest using transparent masks; however, we have found that consideration needs to be given to shorter durations of use to prevent frosting and obscuring the lip movements.

We agree with Jena (2020) in that oculesics (study of using eye movement for communication) has become more important with the advent of COVID-19. The eye movements and the brows are connected and together are capable of communicating confusion, surprise, sadness and joy by both the MHCN and the elderly. The MHCN needs to re-discover the value that eye movements have in conveying non-verbal messages during counselling.

After considering all of the above and re-looking at non-verbal communication concealed behind the masked face, we, as MHCNs, need to consider cultural sensitivity of the elderly accessing our services. The non-verbal expressions of empathy differs amongst cultures (Lorié et al. 2017). Empathy can be expressed through eye movements (Lorie et al. 2017). However, eye contact, its duration, direction and attentiveness has significantly different meanings in age groups and cultural groups, but not in gender groups (Jena 2020; Lorié et al. 2017). Cultural influences might result in the MHCN showing greater reliance on voice tone and kinesics to substitute the lesser use of eye contact. Kinesics, for example, hand gestures or head movements, can indicate acceptance, initiate communication or provide recognition (Keutchafo, Kerr & Jarvis 2020), when otherwise a smile might have been used. However, careful consideration needs to be given to hand movements when emphasising non-verbal communication without being disruptive in the process.

Another possible solution to overcome the challenges of the hidden non-verbal facing the MHCN and advance universal health coverage is digital health (WHO 2019). Counselling via online platforms could help mitigate issues of space, distancing, and mask wearing, traditionally associated with face-to-face counselling. The manipulation of online platforms can include video or audio, or both during the counselling sessions. The preference of the latter will depend on the elderly’s emotional comfort, available bandwidth, and their skill in navigating these platforms (Zhou, Rau & Salvendy 2014). We met with resistance from some of the elderly to the use of online counselling; however, interventions have demonstrated its possibility (Jarvis, Padmanabhanunni & Chipps 2019).

The MHCNs need to be reflective of their non-verbal communication styles and critically examine whether, as wearing a mask, there has been a shift in their interactions to a less effective communication style (Padhy et al. 2020), inclusive of power discourses. In addition, it is important that MHCNs consider if they further contribute to the stressors and vulnerability of the elderly through exhibiting ageism. Ageism, as the stigma attached to the elderly’s ability to use technology, has the possibility of inhibiting the MHCN from promoting and developing the protective factor of online skills (Officer et al. 2016).

## Conclusion

The use of non-verbal communication with the elderly in the current pandemic is complex. The wearing of a mask in a context synonymous with the challenges of physical and psychological vulnerability to COVID-19, physical constraints and restraints, and neurocognitive demands adds to the complexity of counselling, risking it becoming a stressor instead of a protective factor. Consequently, the pandemic’s influence on the landscape of non-verbal communication exposes the need for MHCNs to revisit the fundamentals of counselling showing initiative, whilse displaying cultural sensitivity. The adaptation to counselling with a mask is a process aimed at adjusting our practices such that the mask becomes ‘invisible’. The MHCN is responsible for responding with initiatives to the practice challenges created by wearing masks and contributing positively to #flatten_ the_ mental_illness_curve.

## References

[CIT0001] Anderson, K.G., Ramo, D.E. & Brown, S.A., 2006, ‘Life Stress, Coping and comorbid youth: An examination of the Stress-Vulnerability Model for substance relapse’, *Journal of Psychoactive Drugs*, 38(3), 255–262. 10.1080/02791072.2006.10399851 17165368

[CIT0002] Ames, D.L., Daniel, L., Fiske, S.T. & Todorov, A., 2011, ‘Impression formation: A focus on others’ intents’, in J. Decety and J.T. Cacioppo (eds.), *The Oxford handbook of social neuroscience*, pp. 419–433, Oxford University Press, New York, NY.

[CIT0003] Carbone, S.R., 2020, ‘Flattening the curve of mental ill-health: The importance of primary prevention in managing the mental health impacts of COVID-19’, *Mental Health & Prevention* 19, 200185. 10.1016/j.mhp.2020.200185 32566473 PMC7255235

[CIT0004] CDC, 2020, ‘Coronavirus disease 2019 (Covid-19)’, in *Older adults*, viewed 02 October 2020, from https://www.cdc.gov/coronavirus/2019-ncov/need-extra-precautions/older-adults.html 10.1002/agm2.12114 PMC7280705 32661509

[CIT0005] Fitzgerald, M.A., 1994, ‘Theories of reflection for learning’, in A. Palmer, S. Burns & C. Bulman (eds.), *Reflective practice in nursing*, pp. 63–84, Blackwell Scientific Publications, Oxford. ISBN 0632035978.

[CIT0006] Galea, S., Merchant, R.M. & Lurie, N., 2020, ‘The mental health consequences of COVID-19 and physical distancing: The need for prevention and early intervention’, *JAMA Internal Medicine* 180(6), 817–818. 10.1001/jamainternmed.2020.1562 32275292

[CIT0007] Jarvis, M.A., Padmanabhanunni, A. & Chipps, J., 2019, ‘An evaluation of a low-intensity cognitive behavioral therapy mHealth-supported intervention to reduce loneliness in older people’, *International Journal of Environmental Research and Public Health* 16(7), 1305. 10.3390/ijerph16071305 PMC6480633 30979042

[CIT0008] Jena, A., 2020, ‘Non-verbal communication across different cultures’, *International Journal of Multidisciplinary Educational Research* 9(5), 169.

[CIT0009] Karimi, H. & Alavi, N.M., 2015, ‘Florence nightingale: The mother of nursing’, *Nursing and Midwifery Studies* 4(2), e29475. 10.17795/nmsjournal29475 26339672 PMC4557413

[CIT0010] Keutchafo, E.L.W., Kerr, J. & Jarvis, M.A., 2020, ‘Evidence of nonverbal communication between nurses and older adults: A scoping review’, *BMC Nursing* 19(1), 1–13. 10.1186/s12912-020-00443-9 32550824 PMC7298765

[CIT0011] Kwame, A. & Petrucka, P.M., 2020, ‘Communication in nurse-patient interaction in healthcare settings in sub-Saharan Africa: A scoping review’, *International Journal of Africa Nursing Sciences* 12, 100198. 10.1016/j.ijans.2020.100198

[CIT0012] Lorié, Á., Reinero, D.A., Phillips, M., Zhang, L. & Riess, H., 2017, ‘Culture and nonverbal expressions of empathy in clinical settings: A systematic review’, *Patient Education and Counseling* 100(3), 411–424. 10.1016/j.pec.2016.09.018 27693082

[CIT0013] Mehta, U.M., Venkatasubramanian, G. & Chandra, P.S., 2020, ‘The “mind” behind the “mask”: Assessing mental states and creating therapeutic alliance amidst COVID-19’, *Schizophrenia Research* 222, 503–504. 10.1016/j.schres.2020.05.033 32425355 PMC7229943

[CIT0014] Nelson, H., Hubbard Murdoch, N. & Norman, K., 2021, ‘The role of uncertainty in the experiences of nurses during the Covid-19 pandemic: A phenomenological study’, *Canadian Journal of Nursing Research* 53(2), 124–133. 10.1177/0844562121992202 33541124

[CIT0015] Officer, A., Schneiders, M.L., Wu, D., Nash, P., Thiyagarajan, J.A. & Beard, J.R., 2016, ‘Valuing older people: Time for a global campaign to combat ageism’, *Bulletin of the World Health Organization* 94(10), 710–710A. 10.2471/BLT.16.184960 27843156 PMC5043217

[CIT0016] Padhy, S.K., Rina, K. & Sarkar, S., 2020, ‘Smile, grimace or grin? Recalibrating psychiatrist-patient interaction in the era of face masks’, *Asian Journal of Psychiatry* 53, 102389. 10.1016/j.ajp.2020.102389 32890982 PMC7451215

[CIT0017] Peel, J.L. & Nolan, R.J., 2015, ‘You can’t start a central line? Supervising residents at different stages of the learning cycle’, *Journal of Graduate Medical Education* 7(4), 536–538. 10.4300/JGME-D-15-00025.1 26692963 PMC4675408

[CIT0018] Schlögl, M. & Jones, C.A., 2020, ‘Maintaining our humanity through the mask: Mindful communication during COVID-19’, *Journal of the American Geriatrics Society* 68(5), E12. 10.1111/jgs.16488 32282056 PMC7262056

[CIT0019] South African Government (SA Gov), 2020, ‘Regulations and guidelines – Coronavirus COVID-19’, viewed 04 July 2021, from https://www.gov.za/covid-19/resources/regulations-and-guidelines-coronavirus-covid-19#dma

[CIT0020] Vaidhyanathan, P., Dadlani, N., Meera, S.S. & Chandra, P.S., 2020, ‘Communication beyond barriers-effective communication with individuals with neuropsychiatric disorders when wearing masks’, *Asian Journal of Psychiatry* 54, 102286. https://doi.org/0.1016/j.ajp.2020.102286 32674066 10.1016/j.ajp.2020.102286

[CIT0021] World Health Organisation (WHO), 2019, *WHO Guideline. Recommendations on digital interventions for health system strengthening*, viewed 09 July 2021, from https://apps.who.int/iris/bitstream/handle/10665/311941/9789241550505-eng.pdf?ua=1 31162915

[CIT0022] World Health Organisation (WHO), 2020, *A guide to WHO’s guidance on COVID-19*, viewed 27 September 2020, from https://www.who.int/news-room/feature-stories/detail/a-guide-to-who-s-guidance

[CIT0023] Xyrichis, A., Wynne, J., Mackrill, J., Rafferty, A.M. & Carlyle, A., 2018, ‘Noise pollution in hospitals’, *BMJ*, 363, k4808. 10.1136/bmj.k4808 30449734

[CIT0024] Zhou, J., Rau, P.L.P. & Salvendy, G., 2014, ‘Older adults’ use of smart phones: An investigation of the factors influencing the acceptance of new functions’, *Behaviour and Information Technology* 33(6), 552–560. 10.1080/0144929X.2013.780637

[CIT0025] Zubin, J. & Spring, B., 1977, ‘Vulnerability; a new view of Schizophrenia’, *Journal of Abnormal Psychology* 86(2), 103–126. 10.1037/0021-843X.86.2.103 858828

